# A pilot evaluation of whole blood finger-prick sampling for point-of-care HIV viral load measurement: the UNICORN study

**DOI:** 10.1038/s41598-017-13287-2

**Published:** 2017-10-20

**Authors:** Sarah Fidler, Heather Lewis, Jodi Meyerowitz, Kristin Kuldanek, John Thornhill, David Muir, Alice Bonnissent, Georgina Timson, John Frater

**Affiliations:** 1 Division of Medicine, Wright Fleming Institute, Imperial College, London, UK; 2 Peter Medawar Building for Pathogen Research, Nuffield Department of Medicine, University of Oxford UK; Oxford NIHR BRC, Oxford, UK; Oxford Martin School, Oxford, UK; 3 Imperial College Healthcare Trust, London, UK; 4 Cepheid Inc., Sunnyvale, California, USA

## Abstract

There is a global need for HIV viral load point-of-care (PoC) assays to monitor patients receiving antiretroviral therapy. UNICORN was the first study of an off-label protocol using whole blood finger-prick samples tested with and without a simple three minute spin using a clinic-room microcentrifuge. Two PoC assays were evaluated in 40 HIV-positive participants, 20 with detectable and 20 with undetectable plasma viral load (pVL) (<20 copies/ml). Using 100 µl finger-prick blood samples, the Cepheid Xpert HIV-1 Viral Load and HIV-1 Qual cartridges were compared with laboratory pVL assessment (TaqMan, Roche). For participants with undetectable viraemia by TaqMan, there was poor concordance without centrifugation with the TaqMan platform with only 40% ‘undetectable’ using Xpert VL and 25% ‘not detected’ using the Qual assay. After a 3 minute spin, 100% of samples were undetectable using either assay, showing full concordance with the TaqMan assay. Defining a lower limit of detection of 1000 copies/ml when including a spin, there was 100% concordance with the TaqMan platform with strong correlation (rho 0.95 and 0.94; p < 0.0001 for both assays). When including a simple microcentrifugation step, finger-prick PoC testing was a quick and accurate approach for assessing HIV viraemia, with excellent concordance with validated laboratory approaches.

## Introduction

Antiretroviral therapy (ART) for the treatment of HIV infection inhibits viral replication and results in the recovery of CD4 T lymphocytes, halting clinical progression^1,2^. Monitoring successful ART is through measurement of plasma HIV-1 viral load, with ‘undetectability’ as the main goal of treatment^3^. Current validated assays require plasma, prepared in laboratory conditions from whole blood samples taken by venepuncture. The vast majority of people living with HIV globally are in resource-limited settings where there is reduced availability of such facilities and complications associated with power supply, sample transportation and cold chain preservation, amongst other issues^4^. Even in settings where resource constraints are not so challenging, there is an inevitable time lag between blood sampling, testing, results and action where required, which is time consuming and costly^5^. Given these limitations, alternative approaches to ART monitoring are critical to maintain successful treatment for all people living with HIV^6^. There is, therefore, a need for simpler point-of-care approaches, for example using whole blood and lower blood volumes, as obtained from needle-prick sampling or even dried blood spots^7^.

A further need for simple, rapid throughput HIV plasma viral load (pVL) monitoring is in clinical trials involving a treatment interruption, where rapid and sensitive screening for rebound viraemia is necessary^8–10^. This need is particularly pertinent to the HIV cure field. Here, increasing numbers of studies explore interventions to reduce the size of the HIV proviral reservoir with primary endpoints often reporting ‘time to pVL rebound’, and requiring frequent and accurate pVL monitoring after treatment interruption (TI)^11–13^. Such regular blood draws – often every 2–3 days - are a substantial inconvenience to participants and may impact recruitment^14^.

We present the first study of a simple whole blood finger-prick analysis using an off-label protocol for GeneXpert Cepheid cartridges. We show that, by including a simple 3 minute clinic-based centrifugation step in a small, portable microcentrifuge, HIV viraemia can be assessed from 100 µl of whole blood from a single finger-prick with excellent concordance with the Roche TaqMan system.

## Methods

### Participants and approvals

UNICORN (Use of Needlestick Investigation to Collate Objective Rebound Notifications) was a study evaluating two point-of-care (PoC) assays in parallel by comparing results with pVL obtained from the COBAS AmpliPrep/COBAS TaqMan HIV-1 Test, version 2.0 (Roche Molecular Diagnostics; Pleasanton, California, USA). Plasma samples were drawn from 20 HIV-positive participants with detectable viraemia, and 20 with undetectable pVL on ART (<20 copies/ml) recruited at St Mary’s Hospital, London. The two PoC cartridges studied were the Cepheid Xpert HIV-1 Viral Load and the HIV-1 Qual. The Cepheid Xpert HIV-1 Viral Load provides a quantitative measure of plasma viral load with a reported lower limit of quantitation of 40 copies/ml from 1 ml plasma (and a limit of detection of 15.3 copies/ml using the Viral Quality Assurance (VQA) Laboratory subtype B standards). The HIV-1 Qual cartridge provides a binary ‘detected’/’not detected’ result from 100 µl whole blood, with a reported limit of detection of 203 HIV RNA copies/ml using VQA standards (and 531 copies/ml from dried blood spots).

The study was approved by the local research ethics committee (London Westminster Research Ethics Committee; Reference Number 16/LO/1238; IRAS/CSP 208516) and all participants provided informed signed consent. All experiments were performed in accordance with relevant guidelines and regulations. Participants were not informed of their GeneXpert results. The company, Cepheid, had no role in the design of the study or in collation or interpretation of the data. Free loan of Xpert IV instruments and cartridges for use in the study were provided by the company as their sole contribution.

### Protocol

Four 100 µl finger-prick samples were taken from each participant as well as a 10 ml venepuncture sample which was sent to the clinical laboratory for validated pVL quantitation using the Roche TaqMan assay on the same day. The lower limit of pVL quantification was defined as < 20 copies HIV RNA/ml using venous blood plasma on the Roche TaqMan assay. Two finger-prick samples were processed on the Cepheid Xpert HIV-1 Viral Load and two on the HIV-1 Qual cartridges. Each finger-prick was taken using the provided minivette (Cepheid) which collected 100 µl of blood. For each pair of samples, one was processed neat and one following a 3 minute spin in a microcentrifuge. Details were as follows:

#### Xpert HIV-1 Viral load Assay

The validated protocol for the Xpert HIV-1 Viral Load assay requires 1 ml of plasma and reports results as HIV RNA copies/ml. Instead, we used a 100 µl whole blood finger-prick sample. For the Xpert HIV-1 Viral Load assay without a spin, the 100 µl blood was added to 900 µl sterile PBS (to make up the required 1 ml volume) and added directly to the cartridge for analysis. The reported VL was corrected by × 10 to account for the dilution. For the Xpert Viral Load assay including a spin, the 100 µl blood was added to a 1.8 ml Eppendorf tube containing 1000 µl PBS. This was then centrifuged in a balanced ultra-compact microcentrifuge (Alpha Laboratories) with a fixed 6000 rpm speed (2000 g) for three minutes. 1000 µl of supernatant was then pipetted into the cartridge directly from the Eppendorf. The final result was corrected × 11 to account for the dilution.

#### Xpert Qual Assay

For the Xpert Qual Assay without a spin the 100 µl blood sample was added to 1000 µl buffer provided with the cartridge, and the assay run according to manufacturer’s instructions. Where the spin was incorporated, the 100 µl blood was first added to 100 µl PBS and centrifuged for 3 minutes. 100 µl supernatant was then removed by pipette and added to the buffer and run, as described above.

### Analysis

Results from the Xpert HIV-1 Viral load were reported as HIV RNA copies/ml, having been corrected for dilution. Results from the Xpert Qual Assay were reported as ‘detected’ or ‘not detected’. Data were analysed for concordance and discordance with the Roche TaqMan assay using Cohen’s kappa for categorical data to determine agreement between assays. Viral load values were compared with the Roche TaqMan assay using non-parametric correlations and Bland-Altman analysis.

## Results

Forty participants were studied. Of these, 36 were male and 4 female. The average age was 39 years (range 18–73). Using the Roche TaqMan assay as a standard, 20 participants were selected with undetectable viraemia on ART (<20 copies/ml plasma) and 20 with detectable viraemia (mean 95,219 copies/ml [range 33–1,514,378]). At the same time as blood was taken for pVL analysis, all forty participants were tested using whole blood 100 µl finger-prick samples with both the Cepheid Xpert HIV-1 Qual and Cepheid Xpert HIV-1 Viral Load cartridges, both with and without the 3 minute benchtop centrifugation step. For the Xpert assays, results were available in 90 minutes. The full dataset is presented in Supplementary Table 1.

### Participants with undetectable viraemia

**Figure 1:**
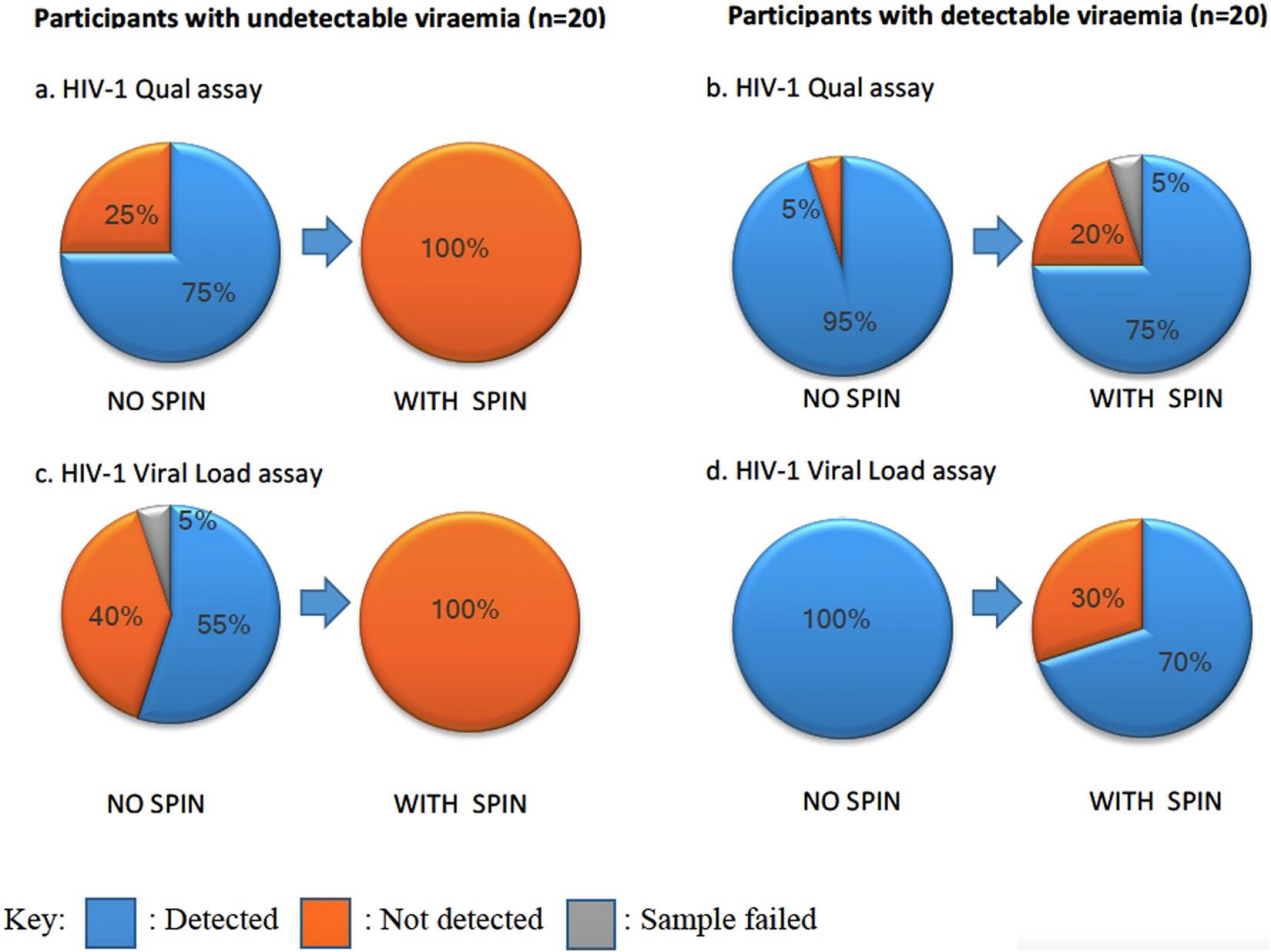
Concordance between Roche TaqMan and GeneXpert assays. Percentages of samples reported ‘detected’ or ‘not detected’ using the qualitative Xpert HIV-1 Qual (**a**,**b**) and quantitative HIV-1 Viral Load cartridges (**c**,**d**), with and without incorporating a 3 minute centrifugation step from a 100 µl whole blood finger-prick sample. Panels a. and c. show participants with undetectable plasma viraemia using the Roche TaqMan assay tested using whole blood finger-prick samples using the GeneXpert Qual and Viral Load cartridges, respectively. Panels b and d show participants with detectable viraemia using the Roche TaqMan assay tested using the Qual and Viral Load cartridges, respectively. Arrows indicate the impact of introducing a 3 minute centrifugation ‘spin’ step to the protocol.

For the 20 participants with undetectable viraemia according to the Roche TaqMan assay, only 5 (25%) and 8 (40%) (1 assay failed) were undetectable according to the Xpert HIV-1 Qual and Viral Load assays, respectively, demonstrating very poor concordance with the Roche TaqMan platform (Fig. 1a,c). Cohen’s kappa value was 0 for both, consistent with poor quality of agreement^15^. This disparity is most likely due to the presence of cellular proviral DNA, which is known to persist in individuals on ART. This suggests that, using standard protocols, whole blood finger-prick analysis is not appropriate for monitoring individuals on ART using the Xpert systems.

We hypothesised that pelleting of infected CD4 T cells by a brief centrifugation step in the clinic room would result in the removal of contaminating proviral HIV DNA. After either a 1 minute or 3 minute spin using a microcentrifuge, no white blood cells were visible on manual inspection using a cell counting chamber (data not shown). We therefore incorporated a 3 minute centrifugation step using a bench-top microcentrifuge into the assay protocol. Following this, all samples (20/20; 100%) were reported as undetectable using both Xpert assays (Fig. 1a,c) suggesting that if a simple bench-top centrifugation step could be included within the protocol, either assay could be used to monitor undetectable viraemia on ART.

### Impact of centrifugation on PoC assay detection of HIV viraemia

Although the 3 minute centrifugation resulted in all samples with undetectable viraemia by Roche TaqMan also being reported undetectable by Xpert, the utility of this step would be negated if sensitivity for detectable viraemia was impacted. We therefore turned to 20 samples from participants with a range of detectable viraemia by Roche TaqMan (mean 95,219 copies/ml; range 33–1,514,378). For these samples, when running the Xpert Qual assay or the Xpert HIV-1 Viral Load assay using the manufacturer’s protocol, 19/20 (95.0%) and 20/20 (100%) were detectable, respectively, showing excellent concordance. (The single sample that failed to amplify for the Qual assay had a pVL of 46 copies/ml by Roche TaqMan).

However, on introducing the centrifugation step only 15/20 (75%) and 14/20 (70%) samples were detectable using the HIV Qual and Viral Load assays respectively (a single sample in the Qual assay with a pVL of 1724 copies/ml by Roche TaqMan resulted in an error), demonstrating that assay sensitivity was negatively impacted by centrifugation. This was supported by a Cohen’s kappa value of 0 for both, consistent with poor quality of agreement^15^. Samples that were reported as undetectable by Xpert had a Roche Taqman pVL of 33, 46, 49 and 158 for the Qual assay and 33, 46, 49, 67, 158 and 269 copies/ml for the HIV-1 Viral Load assay. These data suggest that the lower limit of detection would need to be reviewed if the Xpert platforms with a centrifugation step were to be used for viraemic samples in a PoC setting.

### Fingerprick PoC Xpert analysis with benchtop centrifugation is effective for screening plasma HIV viral loads > 1000 copies/ml

**Figure 2:**
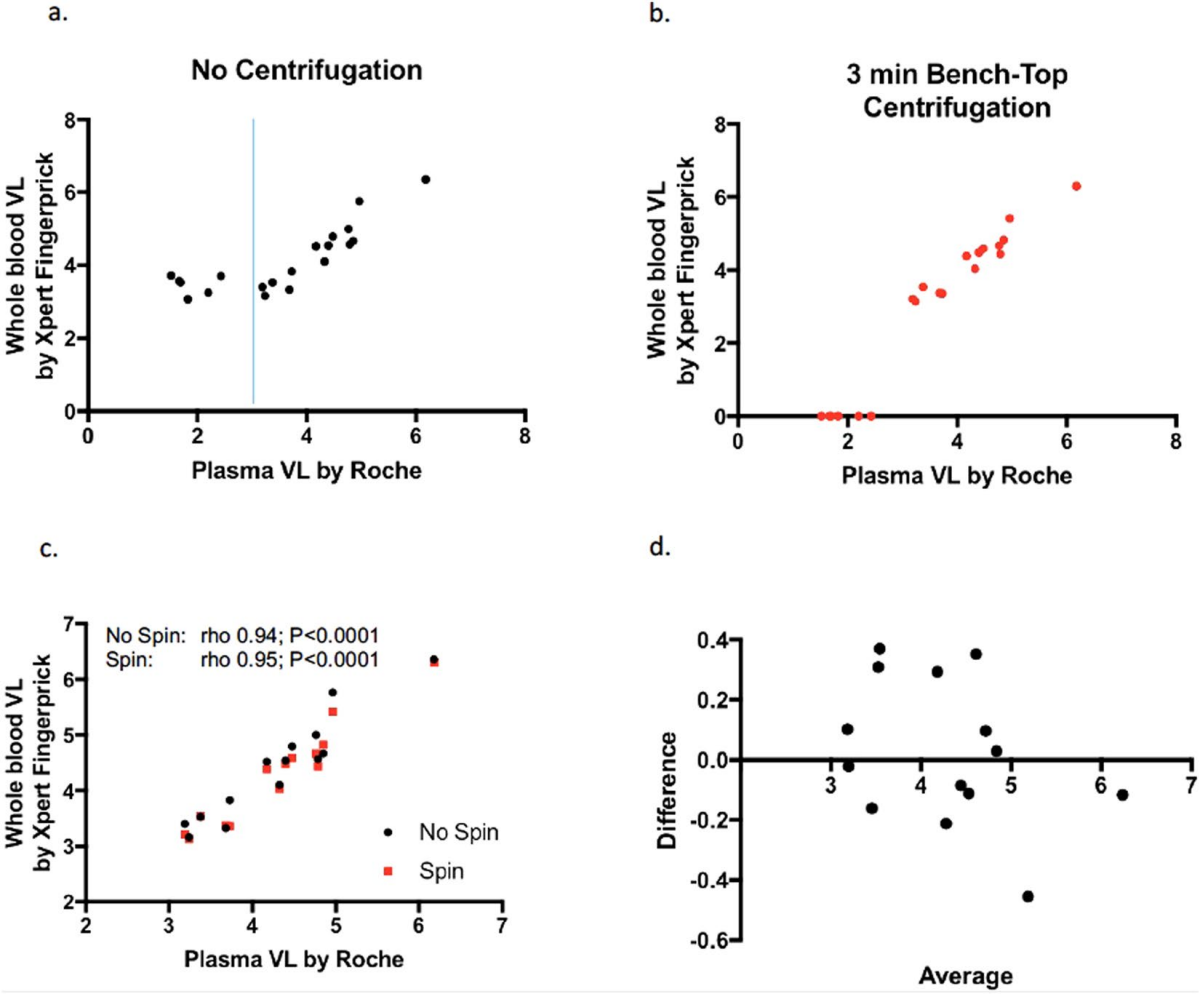
Impact of benchtop centrifugation on PoC finger-prick viral load quantification. Comparison of Roche TaqMan pVL assay with GeneXpert Viral Load assay without (**a**) or with (**b**) a 3 minute centrifugation step. Vertical line in (**a**) shows suggested lower limit of detection for the finger-prick protocol (1000 copies/ml) based on this study. Correlation between Roche TaqMan pVL assay and Xpert Viral Load assays when a lower limit of detection of 1000 copies/ml was imposed and 6 samples with pVL below this excluded (**d**). Values are Spearman’s Rho and P values. Bland Altman analysis comparing the Roche TaqMan with Xpert VL assay with centrifugation for samples > 1000 copies/ml (**d**).

All samples with pVLs by Roche TaqMan > 250 copies/ml were detected as positive by the Xpert Qual assay. There was only one discrepancy between the Xpert Qual and Viral Load platforms with the latter reporting a pVL of 269 copies/ml as undetectable (<40 copies). By proposing a conservative lower limit of detection for the Xpert PoC centrifugation protocol of 1,000 RNA copies/ml, we excluded the 6 samples that were below this value. For the remaining 14 samples the correlation with the Roche TaqMan platform was excellent (rho 0.94; P < 0.0001 and rho 0.95; P < 0.0001 without and with centrifugation, respectively (Spearman’s correlation))(Fig. 2a–c). For quantification above 1000 copies/ml, Bland Altmann analysis revealed excellent concordance between the Roche TaqMan platform and the PoC Xpert whole blood centrifugation protocol, with an average difference between the assays of 0.028 log_10_ copies/ml (95% limits of agreement: −0.45 to 0.50), indicating excellent concordance within clinical requirements (Fig. 2d).

## Conclusion

Finger-prick whole blood sampling could be used to quantify viraemia, potentially facilitating provision of ART monitoring in the field, in resource-limited high-prevalence settings or where a high frequency of testing is required (eg treatment interruption studies). Other studies have reported poor concordance between pVL and PoC whole blood assays^16^. The success of our approach was dependent on the introduction of a straight-forward clinic room 3 minute centrifugation step using an easily portable microcentrifuge. Although we find excellent concordance with samples with undetectable pVL between our protocol and the validated Roche Taqman approach, this is at the expense of a loss of sensitivity for samples with detectable viraemia. Whereas the Cepheid platforms can detect viraemia as low as 15.3 copies/ml and can quantify samples above 40 copies/ml, this is significantly impacted by using lower volumes, using whole blood and incorporating a spin. Although all three adaptations to the protocol might impact sensitivity, we would not expect a 3 minute spin on a microcentrifuge to pellet virions, and the greatest impact is likely to be due to lower sample inputs and the possibility of PCR inhibitors within whole blood. Nevertheless, we propose a limit of sensitivity of 1000 copies/ml pending further optimisation, which is the pVL threshold for detection for viral rebound advocated by the WHO^6^.

The introduction of the micro-centrifuge step can be conducted within the clinic room or in a suitable near-by space, and removes the likely contamination from cellular proviral HIV DNA which otherwise negates the use of whole blood samples. These microcentrifuges are small, self-contained, easily available and portable. The model we used was plugged into the mains electricity supply, although battery-operated devices are also available to facilitate field work. Training required to undertake the spin and remove the supernatant for addition to the Xpert cartridge is very straightforward and could be conducted using disposable plastic pastettes, excluding the need for expertise with laboratory pipettes. Results are available within 90 minutes and there is no requirement for a formal validated laboratory or blood preparation facility, although regular calibration (e.g. yearly) of the Cepheid Xpert machine would be required to guarantee reproducibility and accuracy. The effectiveness of the microcentrifugation step at removing cellular proviral HIV DNA suggests that there could also be a role for this in the preparation of dried blood spots which could significantly impact viral load assessments in resource limited regions, although a clear analysis of the impact on assay sensitivity would need to be considered. Of note, the use of dried blood spots for the analysis of pVL has documented issues around sensitivity and proviral DNA contamination^17^. We did not test centrifuged finger-prick samples in other viral load systems, although there is no obvious reason why these results would not translate to other platforms. We did not formally assess the impact of HIV subtype on the outcome of this protocol as we would not expect the sample preparation steps to impact one HIV subtype over another. Any subtype-associated bias should be unchanged from that within the Cepheid system which is documented elsewhere. Although our sample size was not large enough for a formal assessment, subtype data were available from 23 of the 40 participants and showed a broad range including A, B, C, F, AE and AG/G (Supplementary Table 1), with no evidence of any bias towards success or failure with a specific subtype using our protocol.

With the increasing requirement for widely available pVL monitoring in resource-limited environments as well as high frequency testing as part of treatment interruption studies, there is an urgent need for faster, more accessible pVL testing. We tested both quantitative and qualitative systems. The latter are potentially of interest as, in many cases, the information required for clinical monitoring is the knowledge that pVL remains undetectable and a quantitative value is not necessary. In our hands there was some evidence that the qualitative assay was more sensitive than the HIV-1 VL quantitative cartridge although this would need to be formally tested, as this varies from the company documentation which does not consider the impact of centrifugation or sample dilution. If this was the case, and there was a significant cost-saving in one approach over the other, there might be a strong rationale to consider a simple qualitative approach.

The ultimate goal is widely available rapid and cheap PoC plasma HIV viraemia testing for all. This pilot study shows that this can be achieved from finger-prick sampling with currently available technology. Further larger studies are now needed for a full validation of this approach.

## Electronic supplementary material

Supplementary Information
