# Supplementary material for: A pilot evaluation of whole blood finger-prick sampling for point-of-care HIV viral load measurement: the UNICORN study
Source: Sci Rep. 2017 Oct 20;7:13658. doi: 10.1038/s41598-017-13287-2 (PMC5651802; doi:10.1038/s41598-017-13287-2)
Supplement: Supplementary file 1 — Supplementary Information [file 41598_2017_13287_MOESM1_ESM.pdf]

Supplementary Material

Title Page

A pilot evaluation of whole blood finger-prick sampling for point-of-care HIV viral load measurement: the UNICORN study

[1]Sarah Fidler

[1]Heather Lewis

[2]Jodi Meyerowitz

[1]Kristin Kuldanek

[1]John Thornhill

[3]David Muir

[4]Alice Bonnissent

[4] Georgina Timson

[2]John Frater

[1]Division of Medicine, Wright Fleming Institute, Imperial College, London

[2]Peter Medawar Building for Pathogen Research, Nuffield Department of Medicine, University of Oxford UK; Oxford NIHR BRC, Oxford, UK; Oxford Martin School, Oxford, UK

[3]Imperial College Healthcare Trust, London, UK

[4]Cepheid Inc., Sunnyvale, California, USA

Corresponding Author: John Frater, Peter Medawar Building for Pathogen Research, Nuffield Dept of Medicine, University of Oxford, UK. Email: john.frater@ndm.ox.ac.uk

Supplementary Table 1. Full Dataset for all 40 participants

	**HIV-1 Subtype**	**Roche**	**GeneXpert VL**		**GeneXpert Qual**	
**SPIN step:**			**UNSPUN**	**SPUN**	**UNSPUN**	**SPUN**
**Participant**						
**1**	D/AE	<20	<40	ND	D	ND
**2**	B	<20	<40	ND	D	ND
**3**		<20	*	ND	D	ND
**4**	AG/G	<20	422	<40	D	ND
**5**	AG	<20	<40	ND	ND	ND
**6**	B	<20	<40	ND	ND	ND
**7**		<20	ND	ND	ND	ND
**8**		<20	133	ND	D	ND
**9**		<20	<40	ND	D	ND
**10**		<20	205	ND	D	ND
**11**		<20	193	ND	D	ND
**12**		<20	<40	ND	ND	ND
**13**	AG	<20	172	<40	D	ND
**14**		<20	298	ND	D	ND
**15**		<20	93	ND	D	ND
**16**	A	<20	755	ND	D	ND
**17**	B	<20	52	ND	D	ND
**18**		<20	98	ND	D	ND
**19**	B	<20	122	ND	D	ND
**20**		<20	ND	ND	ND	ND
**21**	B	33	5210	<40	D	ND
**22**		46	3690	<40	ND	ND
**23**	A	49	3390	<40	D	ND
**24**	B	67	1180	<40	D	D
**25**		158	1770	<40	D	ND
**26**	C	269	5040	<40	D	D
**27**		1541	2520	1617	D	D
**28**		1724	1460	1364	D	*
**29**	B	2382	3380	3454	D	D
**30**	B	4792	2130	2354	D	D
**31**		5335	6760	2277	D	D
**32**	B	14857	33300	24200	D	D
**33**	A	21257	12700	10824	D	D
**34**	C/AE	25099	35000	30580	D	D
**35**		30019	62100	38830	D	D
**36**	B	58318	99200	46750	D	D
**37**	B	61479	37200	27390	D	D
**38**	B	71063	46700	66550	D	D
**39**	F	91512	574000	260700	D	D
**40**	C	1514378	2270000	1980000	D	D

Supplementary Table 1. Full Dataset for all 40 participants.

For each of the 40 participants the pVL by TaqMan (Roche), the result of the Xpert Viral Load assay (with and without centrifugation: 'UNSPUN' / 'SPUN') and the result of the Xpert Qual assay (with and without centrifugation: 'UNSPUN' / 'SPUN') is given.

*No result/assay failed. All values are HIV RNA copies/ml, and are corrected for dilution factor. D is 'detected'; ND is 'not detected'.
